# Keratoconus enlargement as a predictor of keratoconus progression

**DOI:** 10.1038/s41598-021-00649-0

**Published:** 2021-10-26

**Authors:** Ana Maria Cunha, Paulo Jorge Correia, Hélio Alves, Luís Torrão, Raúl Moreira, Fernando Falcão-Reis, João Pinheiro-Costa

**Affiliations:** 1 Department of Ophthalmology, Centro Hospitalar Universitário São João, Porto, Portugal; 2 Centro Hospitalar de Vila Nova de Gaia/Espinho, Vila Nova de Gaia, Portugal; 3 Department of Biomedicine, Faculty of Medicine of University of Porto, Porto, Portugal; 4 Department of Surgery and Physiology, Faculty of Medicine of University of Porto, Porto, Portugal; 5 Department of Ophthalmology of São João Hospital, Avenida Prof. Hernâni Monteiro, 4202 – 451 Porto, Portugal

**Keywords:** Predictive markers, Corneal diseases, Eye manifestations

## Abstract

Numerous approaches have been designated to document progression in keratoconus, nevertheless there is no consistent or clear definition of ectasia progression. In this present study, we aim to evaluate Keratoconus Enlargement (KCE) as a parameter to document ectasia progression. We define KCE as an increase of more than 1D in the anterior curvature of non-apical corneal areas. We have designed a longitudinal study in 113 keratoconic eyes to assess keratoconus progression. KCE was compared with variables commonly used for detection of keratoconus progression like Kmax, Km, K2, PachyMin, D-Index, Corneal Astigmatism and PRC of 3.0 mm centered on the thinnest point. The variations of keratometric readings, D-index and ELEBmax showed positive associations with KCE. Evaluating the performance of Kmax, D-index and KCE as isolated parameters to document keratoconus progression we found a sensitivity of 49%, 82% and 77% and a specificity of 100%, 95% and 66% to detect keratoconus progression (p < 0.001 for all). This difference in sensitivity can be explained by the changes in keratoconus outside the small area represented by Kmax. The inclusion of KCE should be considered in the evaluation of keratoconus progression in conjunction with other variables to increase the reliability of our clinical evaluation.

## Introduction

Keratoconus (KC) is a chronic corneal ectasia characterized by a progressive stromal thinning and several structural changes, frequently bilateral and asymmetrical, that causes protrusion and corneal scarring with loss of vision and astigmatism. Subclinical stages of the disease typically have negligeable or no symptoms but in advanced stages there is an important impact on patient’s quality of life^1–4^.

The initial presentation of keratoconus usually happens during puberty, followed by a period of progression of the disease and stabilizes around the fourth decade of life. The risk of progression and progression rate are higher in children and adolescents younger than 19 years and decline considerably after this age^5–7^. Despite the increasing knowledge about the pathophysiology of keratoconus, there is still no curative treatment and it remains one of the most important indicators for corneal transplantation^8–10^. The main goal in the management of keratoconic eyes is to stop disease progression and that is possible with crosslinking to increase the degree of stiffness of the cornea. Cross-linking is recommended for progressive keratoconus at any stage^11–15^. Treatment modalities like spectacles, rigid contact lenses and insertion of corneal ring segments are commonly applied to improve visual function but cannot stop ectasia progression^16^. Therefore, it is very important to know what patients will progress to apply cross-linking early and to prevent more severe stages of the disease^17^.

For those reasons, finding a standardized method to document progression is essential and nowadays there is still no clear definition for this. Global Consensus on keratoconus and ectatic diseases (2015) defined keratoconus progression by a consistent change in at least two of the following parameters: steepening of the anterior corneal surface; steepening of the posterior corneal surface; thinning and/or an increase in the rate of corneal thickness change from the periphery to the thinnest point. The consensus panel also pointed out that a more specific definition of progression was hampered by the scarcity of existing quantitative data and that this information would likely be technology specific^16^.

Evaluation of corneal topography is considered the best method for early detection and monitoring of keratoconus progression and the Pentacam tomography system seems to be the most sensitive method^18,19^. Changes in keratoconic corneas are very complex. Efforts have been done to find the most reliable variables to explain progression but the majority reflect changes in the central cornea or the steepest point in corneal curvature^2^. However, it is possible to document changes in the curvature of the cornea out of the steepest point.

In clinical practice, the authors identified that some patients, despite not meeting progression criteria such as Kmax or pachymetry, had changes in the anterior curvature maps that could mean progression. Thus, we define keratoconus enlargement (KCE) as an increase of more than 1D in the anterior corneal curvature of non-apical area, out of the steepest point represented by Kmax.

Our purpose is to evaluate KCE as a parameter to document ectasia progression, and to compare it to the most recent and reliable parameters used on keratoconus progression.

## Results

A total of 113 eyes of 76 KC patients met the inclusion criteria and were included in this study. A sample characterization of patients is presented in Table 1.

**Table 1:** Summary of sample characterization of patients and characterization of tomographic indices.

	Mean/Median (SD/IQR)	Minimum	Maximum	95% CI
Women, n	n = 26 (23%)			
Right eye, n	n = 50 (44.2%)			
Age, years	24.09 (3.93)	14	30	23.36–24.82
SCVA, decimal*	0.70 (0.50)	0.10	1.00	0.62–0.73
BCVA, decimal*	0.90 (0.20)	0.10	1.00	0.80–0.87
Spherical equivalent, diopters*	− 1.50 (2.66)	− 9.25	0.75	− 2.53 to − 1.71
Kmax, diopters	55.07 (7.00)	42.40	78.00	53.76–56.37
ΔKmax	0.43 (1.34)	− 2.80	5.90	0.16–0.68
Km, diopters	47.50 (4.67)	39.90	66.40	46.62–48.36
ΔKm	0.35 (0.59)	− 1.00	2.20	0.22–0.48
K1, diopters	45.90 (4.35)	39.20	63.60	45.09–46.71
ΔK1	0.31 (0.57)	− 0.80	2.40	0.20–0.41
K2, diopters	49.25 (5.25)	40.70	69.40	48.27–50.23
ΔK2	0.39 (0.80)	− 1.70	2.90	0.24–0.54
ISV	84.20 (39.02)	16	185	76.93–91.48
ΔISV	2.04 (7.47)	− 20	55	0.64–3.43
ARTmax	176.33 (79.76)	44	518	161.46–191.19
ΔARTmax	− 5.82 (25.17)	− 146	60	− 10.51 to − 1.13
PachyMin, μm	461.26 (46.20)	338	574	452.61–469.91
Δ PachyMin	− 0.63 (10.94)	− 32	36	− 2.68 to − 1.41
D-Index	8.33 (4.47)	0.81	24.22	7.50–9.16
Δ D-Index	0.39 (0.74)	− 1.22	2.97	0.25–0.53
ELEBmax, μm	59.55 (26.85)	11	164	54.33–64.77
ΔELEBmax	2.61 (7.06)	− 44	20	1.23–3.98
PCR, mm	5.07 (0.66)	3.39	6.52	4.95–5.20
ΔPCR	− 0.07 (0.10)	− 0.57	0.25	− 0.09 to − 0.05
Astig, diopters	3.35 (2.15)	− 0.8	10.6	2.95–3.75
ΔAstig	0.09 (0.67)	− 1.8	2.3	− 0.04 to − 0.21

Results are expressed as mean ± SD for continuous variables (*results expressed as median ± IQR), 95% confidence intervals (95% CI) and female gender and right eyes are expressed as count and percentage.

*SCVA* spectacle corrected visual acuity, *BCVA* best corrected visual acuity, *Kmax* maximum keratometry, *Km* mean keratometry, *K1* keratometry of flat meridian, *K2* keratometry of steepest meridian, *ISV* index of surface variance, *ARTmax* Ambrósio relational thickness maximum, *PachyMin* minimum pachymetry, *D-Index* Belin/Ambrósio D-Index, *ELEBmax* maximum elevation of corneal back surface, *PCR* posterior radius of curvature from the 3.0 mm centered on the thinnest point, *Astig* corneal astigmatism. Δ represents the variations of parameter readings between the first and the second measurement after 12 ± 3 months.

The mean age was 24.09 (s.d. 3.93) years and individuals were predominately men (23% women). Best corrected visual acuity (BCVA) was 0.90 ± 0.20, with glasses or contact lenses. The mean spherical equivalent of the studied eyes was − 1.50 ± 2.66 diopters. Twenty-five patients had a documented history of atopy (allergic asthma, atopic dermatitis, allergic rhinitis) or eye rubbing.

A characterization of mean tomographic values at baseline and the difference among the first and the second measurement after 12 ± 3 months is presented in Table 1. Regarding keratoconus classification, the majority of eyes were classified as stage 2 (n = 24; 21.2%) or stage 3 (n = 25; 22.1%). Eyes in all stages of keratoconus were included except stage 4. The number of eyes that would be classified as progressors taking into account single tomographic parameters (Kmax, Km, Pachymin, D, Astig, K2, PCR) is shown in Table 2.

**Table 2:** Absolute and relative frequencies of keratoconus progressing eyes when considering each progression parameter alone.

Variable	Progressors n (%)
Kmax	28 (24.8%)
Km	26 (23.0%)
Pachymin	28 (24.8%)
D-Index	50 (44.2%)
Astig	10 (8.8%)
K2	25 (22.1%)
PCR	45 (39.8%)

*Kmax* maximum keratometry, *Km* mean keratometry, *PachyMin* minimum pachymetry, *D-Index* Belin/Ambrósio D-Index, *Astig* corneal astigmatism, *K2* keratometry of steepest meridian, *PCR* posterior radius of curvature from the 3.0 mm centered on the thinnest point.

In regard to KCE, progression was considered to be present in 63 eyes (55.8%) comparing the first and the second measurements, whereas 50 eyes (44.2%) did not show area progression. Table 3 presents the variation of different parameters analyzed in the groups with and without KCE. The variations of keratometric readings (ΔKmax, ΔKm, ΔK1, ΔK2) showed positive associations with area progression (p = 0.001 for ΔKmax and p < 0.001 for ΔKm, ΔK1, ΔK2). The variation of D-index and ELEBmax also increased significantly in the group that showed KCE (ΔD = 0.56, p = 0.005 and ΔELEBmax = 6.217, p = 0.008). The remaining analyzed parameters (ΔISV, ΔARTmax, ΔPachymin, ΔPCR, ΔAstig) did not show significant differences between groups.

**Table 3:** Variation of different progression parameters between measurements (12 ± 3 months apart) in groups with or without KCE progression.

	No progression in KCE (n = 50)		Progression in KCE (n = 63)		P value
ΔKmax (mean, SD)	− 0.02	1.019	0.78	1.467	**0.001**
ΔKm (median/IQR)	− 0.10	0.30	0.50	0.70	** < 0.001**
ΔK1 (mean, SD)	− 0.05	0.251	0.59	0.593	** < 0.001**
ΔK2 (mean, SD)	− 0.07	0.503	0.76	0.803	** < 0.001**
ΔISV (mean, SD)	0.64	3.439	3.14	9.411	0.054
ΔARTmax (median/IQR)	− 4.00	35.00	− 3.00	27.00	0.178
ΔPakmin (mean, SD)	1.61	9.855	− 2.02	11.205	0.073
Δ D-Index (mean, SD)	0.19	0.473	0.56	0.867	**0.005**
ΔELEBmax (mean, SD)	1.54	3.233	4.06	6.217	**0.008**
ΔPCR (mean, SD)	− 0.05	0.066	− 0.08	0.126	0.152
ΔAstg (mean, SD)	− 0.02	0.431	0.17	0.813	0.121

*KCE* keratoconus enlargement, *Kmax* maximum keratometry, *Km* mean keratometry, *K1* keratometry of flat meridian, *K2* keratometry of steepest meridian, *ISV* index of surface variance, *ARTmax* Ambrósio relational thickness maximum, *PachyMin* minimum pachymetry, *D-Index* Belin/Ambrósio D-Index, *ELEBmax* maximum elevation of corneal back surface, *PCR* posterior radius of curvature from the 3.0 mm centered on the thinnest point, *Astig* corneal astigmatism, Δ represents the variations of parameter readings between the first and the second measurement after 12 ± 3 months.

Considering KC progression as a significant evolution in at least 2 tomographic variables, 57 eyes (50.4%) showed progression, of which 36 eyes (31.9%) progressed in 3 or more variables simultaneously. Forty-four eyes (38.9%) did not progress in any variable and 12 eyes (10.6%) were considered as non-progressive as they only progress in one variable.

Regarding Kmax, 28 eyes showed progression in this parameter and all of them (100%) showed progression in at least 2 other variables. Of the 85 eyes that did not show progression in Kmax, 29 (34.1%) progressed in at least 2 other parameters. The performance of kmax, D-index and KCE as isolated predictors of KC progression are presented in Table 4. Evaluating the performance of Kmax alone as a predictor of KC progression (defined as a change in two or more variables), we found a sensitivity of 49%, a specificity of 100%, a PPV of 100% and NPV of 66% (p < 0.001 for the association of Kmax and KC progression).

**Table 4:** The performance of Kmax, D-index and KCE as isolated predictors of KC progression (defined as a significant change in two or more variables).

	Sensitivity (%)	Specificity (%)	PPV (%)	NPV (%)
Kmax	49	100	100	66
D-index	82	95	94	84
KCE	77	66	70	74

*Kmax* maximum keratometry, *D-Index* Belin/Ambrósio D-Index, *KCE* Keratoconus Enlargement, *PPV* positive predictive value, *NPV* negative predictive value.

As far as the D-Index is concerned, of the 50 eyes that showed progression, only 3 did not show progression in 2 or more other parameters. Ten (15.9%) of the 63 eyes that did not progress in D-index showed progression in at least two other parameters. This index presented a sensitivity of 82%, a specificity of 95%, a PPV of 94% and NPV of 84% when used isolatedly to detect progression (p < 0.001). Regarding KCE, 63 eyes showed progression in this parameter. Within these, 19 eyes (30.2%) did not progress in other 2 parameters. Of the 50 eyes whose keratoconus did not enlarge, 13 eyes (26%) progressed in 2 or more parameters. A sensitivity of 77%, a specificity of 66%, a PPV of 70% and NPV of 74% were demonstrated for the use of KCE alone as a 207 marker of KC progression (p < 0.001).

## Discussion

We wanted to evaluate the performance of KCE to document keratoconus progression. The authors defined KCE as an increase of more than 1D in the anterior curvature of non-apical corneal areas, out of the small area represented by Kmax. We compared KCE with other studied non-validated variables to document progression^2^.

Kmax is commonly used as an indicator of ectatic progression. Some studies define progression as an increase in Kmax but there is no consensus on which cut-off would be more appropriate (0.75 D vs 1D)^20,21^. Kmax characterizes the steepest anterior corneal curvature from a small area and fails to reproduce changes that occur in other areas of anterior cornea, posterior cornea and pachymetry. Moreover, progression of keratoconus can occur with no change of Kmax^22,23^, like we saw in 29 eyes that progressed in two or more variables without progression in Kmax. We found that the sensitivity of Kmax alone to detect keratoconus progression was lower than KCE (49% vs 77% sensitivity) when we consider progression as change in two or more variables. This finding can be explained by changes in keratoconus out of the small area represented by Kmax. However, when Kmax changes more than 1D, keratoconus ectasia always progresses (100% PPV).

Moreover, despite KCE can detect progression in Kmax (only 4 eyes showed a progressing Kmax without progression in KCE), the opposite is not true (39 of 63 eyes with enlargement in keratoconus had no change in Kmax). This may mean that KCE can be an earlier marker of progression than Kmax. In clinical practice we recognize that some eyes suffer an enlargement of keratoconus with no or minimal changes in the small area represented by Kmax.

Additionally, we found significant differences in corneal curvature parameters Kmax, Km, K1 and K2 between eyes with and without KCE. Choi et al. found that K1, K2 and Km have significantly different change rates in non-progressing and progressing keratoconic eyes^24^ which supports the sensitivity of our variable to detect progression. K1, K2 and Km are well positioned to detect progression in keratoconus^21^.

As far as pachymetry is concerned, a decrease of 2% in anual central corneal thickness (CCT) has been shown to represent progression of the pathology^2^, but these anual changes appear no to differ significantly between non-progressing and progressing eyes^24^. CCT and thinnest corneal thickness (TCT) were previously identified as having a suboptimal performance regarding reproducibility^21^ and we found no significant differences in pachymetry between groups with KCE. Duncan et al. tested three tomographic parameters in normal eyes: corneal thickness at the thinnest point, anterior and posterior radius of curvature (ARC, PRC) from the 3.0 mm optical zone centered on the thinnest point and concluded that they may be good indicators of early progression of the disease. They determined the 95% one-sided confidence interval for each variable, since progression is showed by thinning and/or steepening of the anterior and/or posterior corneal surfaces. Those 95% CI were quite narrow for all parameters (7.88 μm for corneal thickness, 0.024 mm for ARC and 0.083 mm for PRC), indicating that greater changes in these parameters may indicate progression^2^. We found no significant differences in PRC between groups of KCE progression. Early ectatic changes usually affect the posterior corneal surface before they become evident on the anterior surface^25,26^. Kitazawa et al., describe the imbalance of the anterior and posterior corneal surface area in keratoconic eyes at the early stage of the disease^27^.

Moreover, ELEBmax demonstrated a good relation with KCE which can corroborate our hypothesis that KCE can be an early marker of keratoconus progression. Kanellopoulos et al. analyzed several anterior surface pentacam-derived topometric indices (ISV, IVA, KI, CKI, IHA, IHD, Rmin) and concluded that ISV and the index of height decentration (IHD) could be the most sensitive and specific indices in the diagnosis and progression of keratoconus^28^. Shajari et al. tested some variables: D-index, ISV, IHA, KI and KPI (KPI—Keratoconus Progression Index—created with a logistic regression analysis, using Pachymin, PRC and ELEBmax) using ROC analysis and found that D-index and KPI are the parameters with higher sensitivity and specificity to document keratoconus progression, discarding ISV and IHA as good parameters^29^. We documented significant differences in D-index between groups of KCE progression but no significant differences for ISV.

Visual acuity and manifest refraction are unreliable variables that do not correlate well with keratoconus severity or progression. Many factors may influence visual acuity such as wearing contact lenses or spectacles^30,31^, so the authors decided not to analyze this parameter.

Among the variables that were analyzed, we found that D-index is the most sensitive and specific to document keratoconus progression, which is in agreement with other studies^28^, with a sensitivity of 82% and a specificity of 95%. The second most sensitive variable was KCE (sensitivity of 77%) and finally Kmax (sensitivity of 49%). As a multimetric parameter, D-index (Df—deviation of front surface elevation difference; Db—deviation of back surface elevation difference; Dp—deviation of pachymetric progression; Dt—deviation of thinnest point and Da—deviation of ARTMax/Ambrósio relational thickness maximum), is better to one isolated parameter regarding the evaluation of progression of keratoconus. In some eyes the cornea may become thinner without a change in Kmax, and the opposite may also be true^30^. In the same way, KCE may not document progression in all progressing keratoconic eyes, and that could explain the calculated sensitivity of 77% for progression. D-index was significantly different between eyes with and without enlargement in keratoconus. That fact supports the use of the parameter KCE as a progression marker.

For all the reasons listed here, we suggest to include KCE when evaluating keratoconus progression. It can be an early marker of disease progression and has a good sensitivity when compared with other commonly used parameters to document progression, like Kmax. KCE is a useful and easy tool that helps to detect progression. It can be used in different tomographic systems as long as they allow the evaluation of the anterior axial/sagittal curvature comparative maps. However, we recommend considering KCE together with other variables to increase the reliability of clinical evaluation, insofar as this parameter only evaluates the anterior surface of the cornea. Also, possibly this parameter may be included in the construction of new indices to document disease progression more accurately.

Our study had to face some limitations. First, we used single measurements for all the tested parameters and according to Ivo Guber et al., averaging across several images results in lower level of measurement noise^21^. Although a high repeatability of measurements of the Pentacam system is well documented in healthy eyes, it is much poorer in ectatic, irregular corneas^32^. In our analysis we only included eyes that received an ‘OK’ from the quality check of the Scheimpflug system in order to increase reliability, and so than, a limited number of eyes in the higher stages of the disease were involved. Second, the only method used to evaluate progression was Scheimpflug imaging, and nowadays other technologies, like biomechanical analysis, could also be considered to measure progression^33^. Finally, we are comparing KCE with other non-validated parameters. In those eyes where KCE is the only parameter showing progression, we cannot be sure if they are really progressing the ectasia and if KCE can be an early marker of progression. Research on this topic is growing and we think that the most accurate method of documenting progression is ever closer.

Additional trials for a more accurate definition of keratoconus progression need to be realized. It is possible to retard or even halt progression of keratoconus ectasia with crosslinking^15^ and the concern must be to step in early, before progression of keratoconus to advance to higher stages, decreasing the patient’s quality of life due to the decreased of visual acuity^29^.

## Materials and methods

We have designed a longitudinal study in which 76 patients diagnosed with Keratoconus (KC) in the Department of Ophthalmology of Centro Hospitalar Universitário de São João, Portugal, were analyzed. The study was conducted according to the Helsinki Declaration and had institutional review board approval from Centro Hospitalar Universitário de São João (CHUSJ) ethics committee. A written informed consent was obtained from all individual participants or, if participants are under 16, from a parent and/or legal guardian.

Patients with KC, aged from 14 to 30 years old, followed in our Ophthalmology Corneal Department, were identified and consecutively included between October and December 2018. All selected patients had more than 1 year of follow-up by a corneal specialist (JPC, LT or RM) and at least 3 Scheimpflug tomography measurements (Pentacam HR, OCULUS Optikgeräte GmbH, Wetzlar, Germany). By routine, Scheimpflug images were only acquired if the patients stopped wearing contact lenses at least 48 h prior to measurement. All measurements were performed by trained Orthoptists, and in cases where the automatic image quality check was not labeled with “OK”, the exam was repeated. All stages of KC were incorporated in the study, including eyes with subclinical Keratoconus only with early tomographic alterations (in these cases, the other eye needed to show clear signs of clinical Keratoconus). Only imaging with a quality check resulting in “OK” was involved in this study, to ensure higher reliability of measurements.

We excluded from analysis: KC eyes with previous ocular surgery (corneal crosslinking, corneal rings, corneal transplant); eyes with very progressive disease (corneal thickness at thinnest point < 350um, corneal hydrops or deep corneal scars), as this group consistently failed an “OK” after the internal scan quality check; eyes without tomographic changes suggestive of subclinical KC. We analyzed Scheimpflug scans of patients with more than 1 year of follow-up, with two scans separated by 12 ± 3 months, for evaluation of progression. Both eyes of the same patient were included when they met inclusion criteria.

Variables studied and used for Keratoconus detection and progression analysis, were maximum keratometry (Kmax), minimum pachymetry (PachyMin), mean keratometry (Km), keratometry of flat meridian (K1), keratometry of steepest meridian (K2), corneal astigmatism (Astig = K2 − K1), maximum elevation of corneal back surface (ELEBmax), posterior radius of curvature from the 3.0 mm centered on the thinnest point (PCR), Ambrósio relational thickness maximum (ARTmax), index of surface variance (ISV) and Belin/Ambrósio D-Index (D).

For progression analysis, the authors only used parameters that are commonly accepted as progression markers with described cut-offs (although not validated). Values representing progression of each analyzed parameter are presented in Table 5.

**Table 5:** Keratoconus progression parameters and respective cut-off values used to document progression.

Variable	Cut-off value
Kmax	1D increase
PachyMin	2% decrease
Km	0.75D increase
K2	1D increase
Astig	1D increase
PCR	0.085 mm decrease
D-Index	0.42 increase

*Kmax* maximum keratometry, *PachyMin* minimum pachymetry, *Km* mean keratometry, *K2* keratometry of steepest meridian, *Astig* corneal astigmatism, *PCR* posterior radius of curvature from the 3.0 mm centered on the thinnest point, *D-Index* Belin/Ambrósio D-Index, *D* diopter.

**Figure 1:**
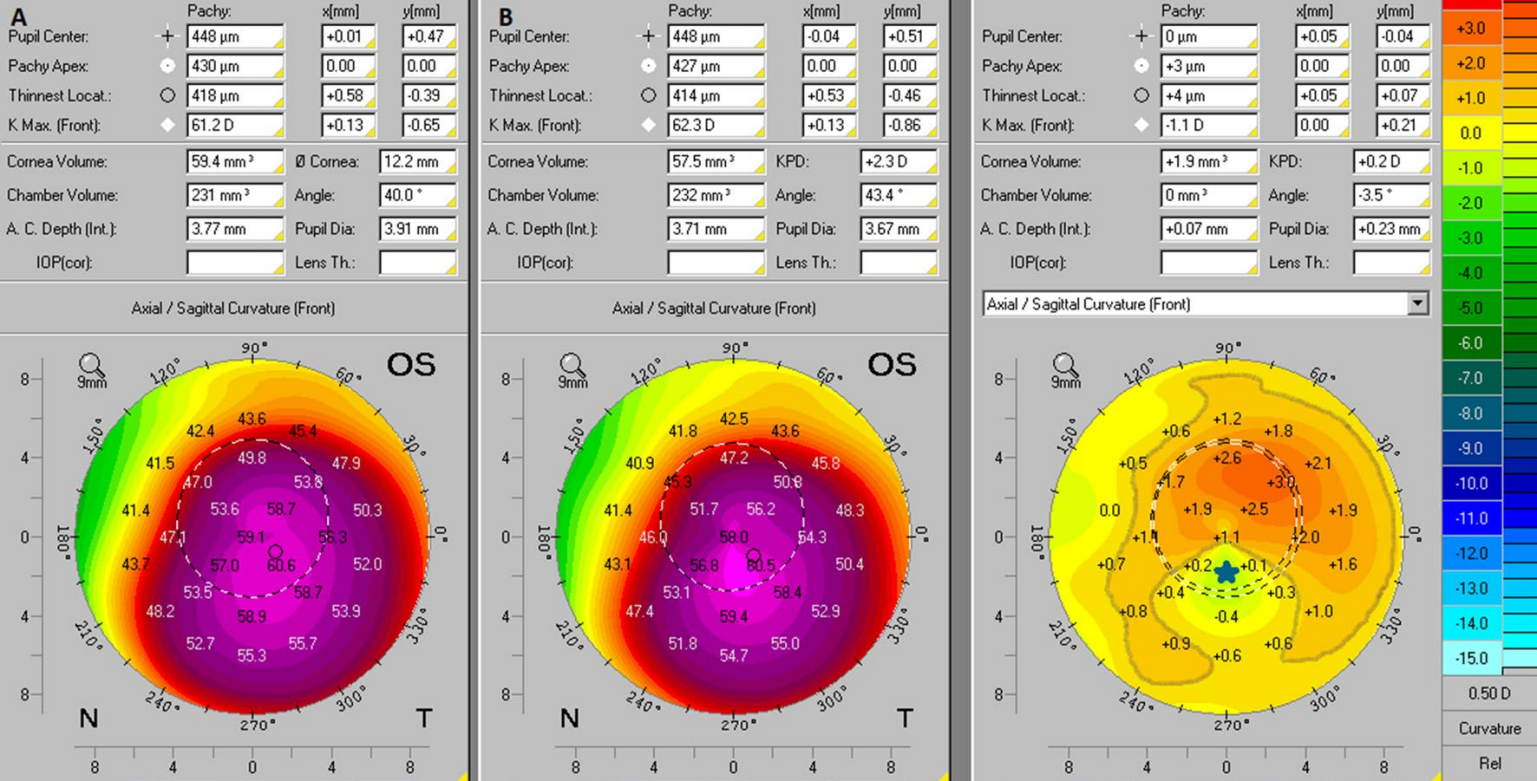
Anterior axial/sagittal curvature comparative maps of Pentacam HR images. In this case, the axial/sagittal curvature maps had an interval of 12 months. In the initial exam (**B**) the Kmax was 62.3D and in the subsequent exam (**A**) the Kmax was 61.2D, so there was no progression in this variable (blue star). On the other way, the comparative map showed an increase of more than 1D in the anterior curvature of the non-apical corneal zone which was highlighted by the gray line, thus showing KCE progression.

**Figure 2:**
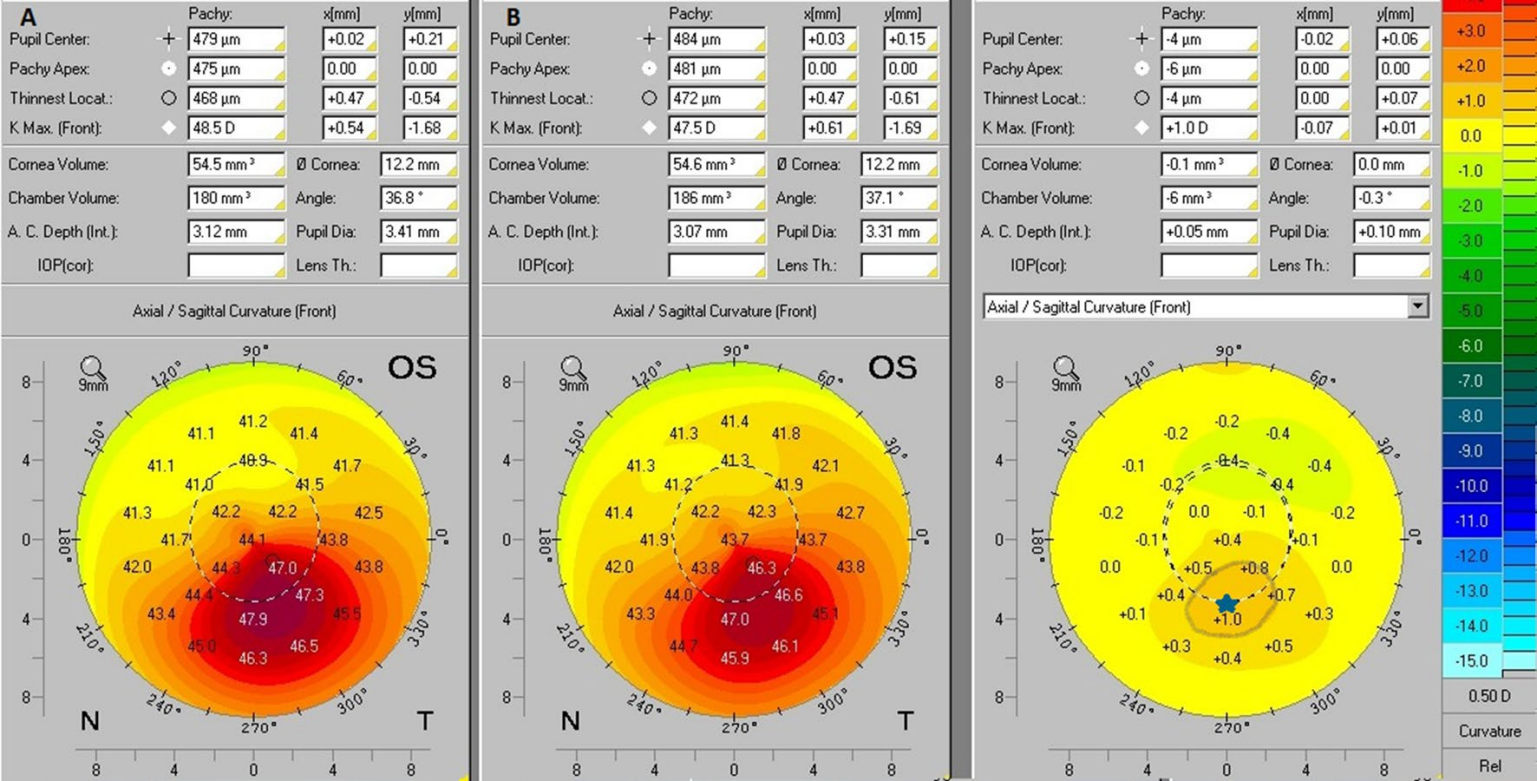
Anterior axial/sagittal curvature comparative maps of Pentacam HR images. In this case, the axial/sagittal curvature maps had an interval of 12 months. In the initial exam (**B**) the Kmax was 47.5D and in the subsequent exam (**A**) the Kmax was 48.5D, so there was progression of + 1D in this variable (blue star). On the other way, the comparative map did not show an increase of more than 1D in the anterior curvature outside the non-apical corneal zone, and therefore there was no KCE progression.

**Figure 3:**
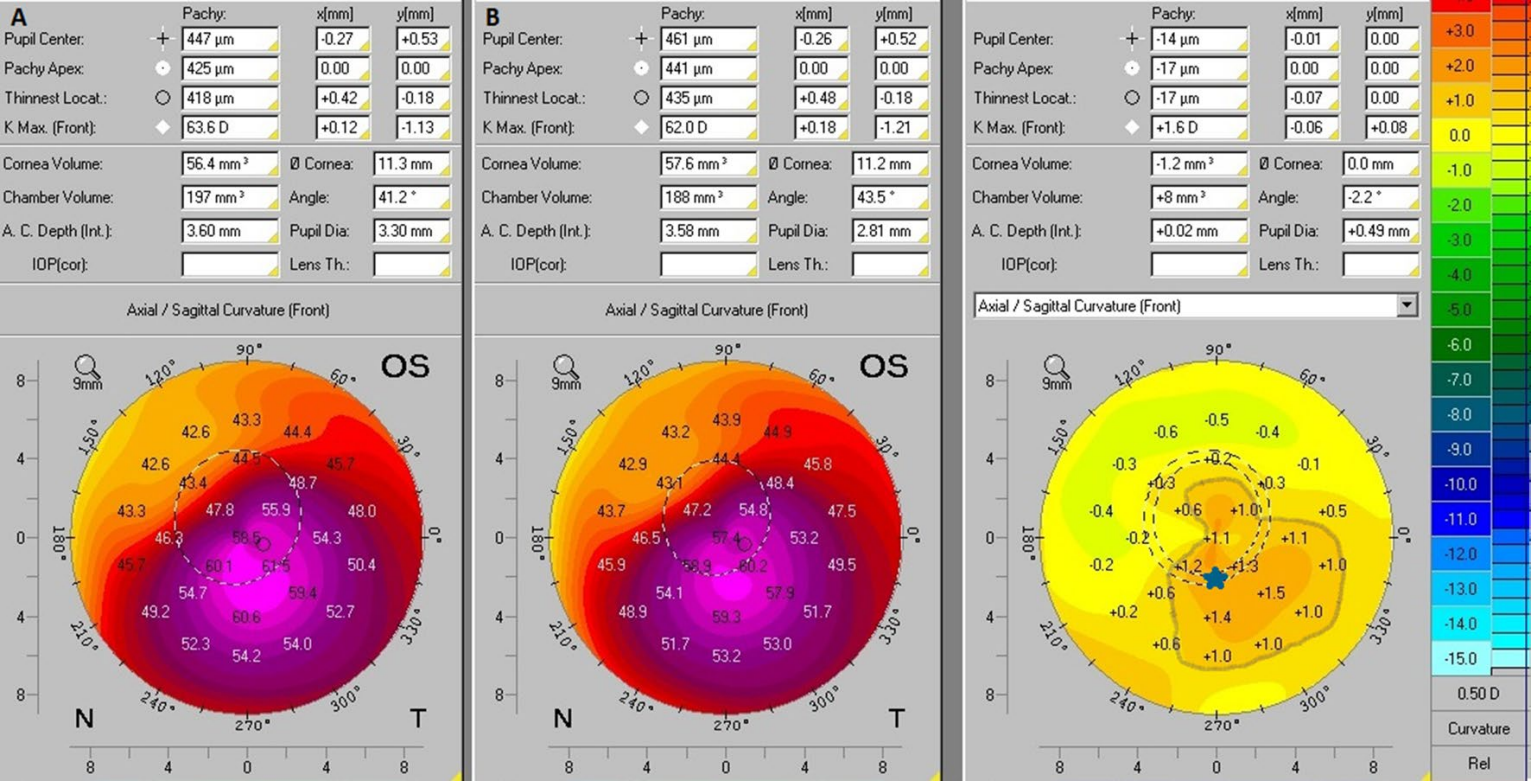
Anterior axial/sagittal curvature comparative maps of Pentacam HR images. In this case, the axial/sagittal curvature maps had an interval of 12 months. In the initial exam (**B**) the Kmax was 62.0D and in the subsequent exam (**A**) the Kmax was 63.6D, so there was progression of + 1.6D in this variable (blue star). Moreover, the comparative map showed an increase of more than 1D in the anterior curvature outside the apical corneal zone, which is highlighted by the gray line, thus showing KCE progression.

The evaluation of KCE was obtained with interpretation of the anterior axial/sagittal curvature comparative maps. KCE was identified when an increase of more than 1D in the anterior curvature comparative maps out of steepest point represented by Kmax, was observed. Three examples of Pentacam HR comparative maps are presented in Figs. 1, 2 and 3. These figures show practical examples of the application of KCE to progression.

Progression of KC was defined when at least two of the studied variables confirm progression. KCE was analyzed simultaneously by two observers (AMC and JPC) in anterior axial/sagital curvature comparative maps of Pentacam HR.

### Statistical evaluation

The sample’s characteristics were summarized and data are exposed as counts and proportions for categorical variables, and as mean and standard deviation (or median and interquartile range, when distributions were skewed) for continuous variables. The prospective variation in keratometric indices was measured subtracting the values at baseline from the second measurement (i.e. a positive delta value indicates an increase in the values of the specific parameter). To evaluate the distribution of keratometric variables across patients classified as progressing or not progressing according to Keratoconus enlargement alone, independent samples *t* tests, Mann–Whitney U and Chi-square tests were used, as suitable. The significance level was set at 0.05. To assess the performance of Kmax, D-index and Keratoconus enlargement as single predictors of progression, we performed Chi-square tests and calculated the positive and negative predictive values (PPV/NPV), the sensitivity and specificity, of each of these variables, taking as reference the classification of progression when at least 2 variables exceeded the defined thresholds (as previously stated). Statistical analysis was used SPSS statistical software package version 24 (SPSS inc., Chicago IL., USA).

### Ethical approval

The current study was performed in accordance with the Declaration of Helsinki.

## Data Availability

The datasets used and analyzed during the current study are available from the corresponding author on request.
